# Supplementary material for: Uncontrolled angiogenic precursor expansion causes coronary artery anomalies in mice lacking Pofut1
Source: Nat Commun. 2017 Sep 18;8:578. doi: 10.1038/s41467-017-00654-w (PMC5603578; doi:10.1038/s41467-017-00654-w)
Supplement: Supplementary file 1 — Supplementary Information [file 41467_2017_654_MOESM1_ESM.pdf]

Description of Supplementary Files

File Name: Supplementary Information
Description: Supplementary Figures and Supplementary Tables.

File Name: Peer Review File

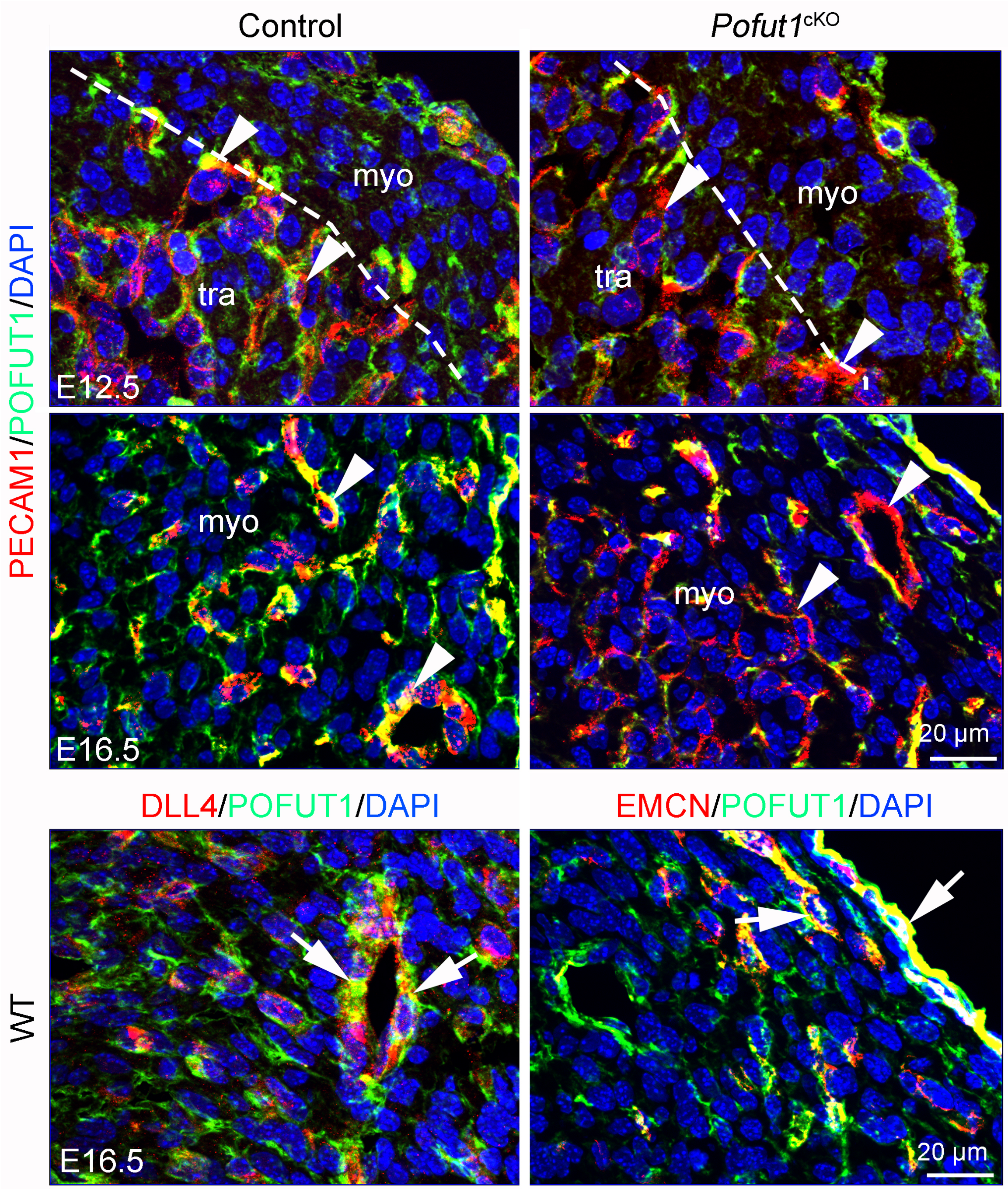

Supplementary Fig. 1. POFUT1 is predominantly expressed in cardiac endocardium and coronary endothelium. Co-immunostaining of POFUT1 with PECAM1 on E12.5 and E16.5 heart sections shows that POFUT1 is highly expressed in cardiac endocardium and coronary endothelium (arrowheads), which is dramatically diminished in *Pofut1*$^{f/f}$;*Nfatc1*^{Cre} (*Pofut1*^{cKO}) embryos. Co-immunostaining of POFUT1 with DLL4 (arterial maker) or EMCN (venous marker) shows that POFUT1 is expressed in both arterial and venous endothelial cells (arrows). tra, trabeculae; myo, myocardium.

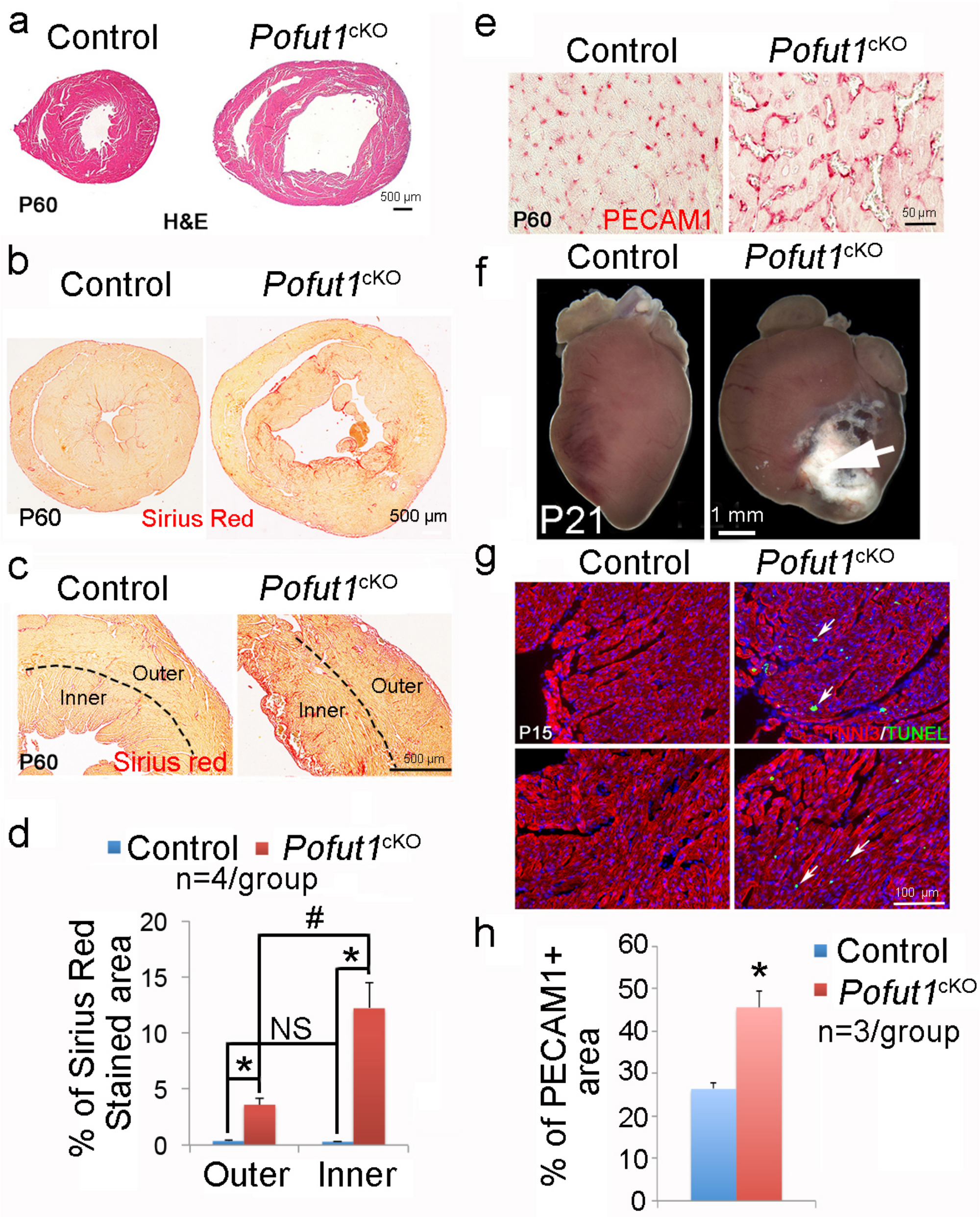

Supplementary Fig. 2. Loss of endocardial *Pofut1* causes cardiac fibrosis, myocardial infarction and increased coronaries. (**a**) HE stained P60 heart sections shows dilated ventricular chamber of *Pofut1*^{cKO} hearts. (**b-d**) Sirius red staining shows *Pofut1*^{cKO} mice have increased fibrosis in the ventricular wall and this increase is more prominent in the inner layer when compares to the outer layer. (**e**) PECAM1 staining shows dilated coronary vessels in two-month old *Pofut1*^{cKO} mice. (**f**) whole mount view of hearts shows myocardial infractions in a representative *Pofut1*^{cKO} mouse dies at P21. (**g**) TUNEL and TNNI3 co-stained heart sections of P15 hearts reveals apoptotic cardiomyocytes (arrows) in *Pofut1*^{cKO} mice. Note that there are few, if any, apoptotic cells in myocardium of control hearts at this stage. (**h**) Quantification of PECAM1 staining shown in Fig. 1j indicates increased coronary density in *Pofut1*^{cKO} mice.

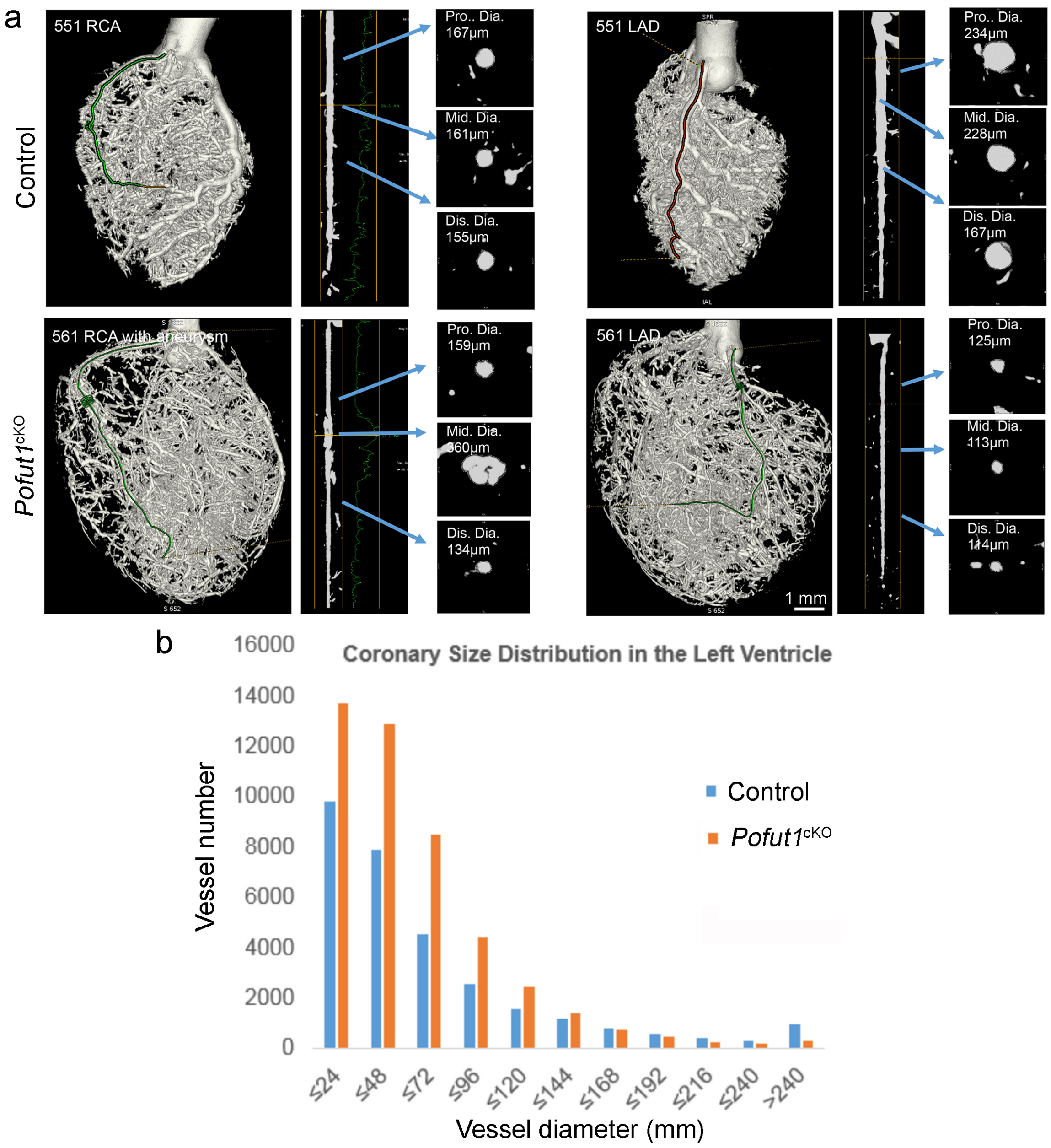

Supplementary Fig. 3. MicroCT analysis of coronary arteries. (**a**) Representative microCT imaging analysis of major coronary arteries of P19 control and *Pofut1*^{cKO} mice indicates underdeveloped main coronary arteries indicated by narrowed lumens and an aneurysm indicated by dilated middle part of the right coronary artery in *Pofut1*^{cKO} heart. (**b**) Quantification of coronary arterial vessels from the same paired hearts in **a**.

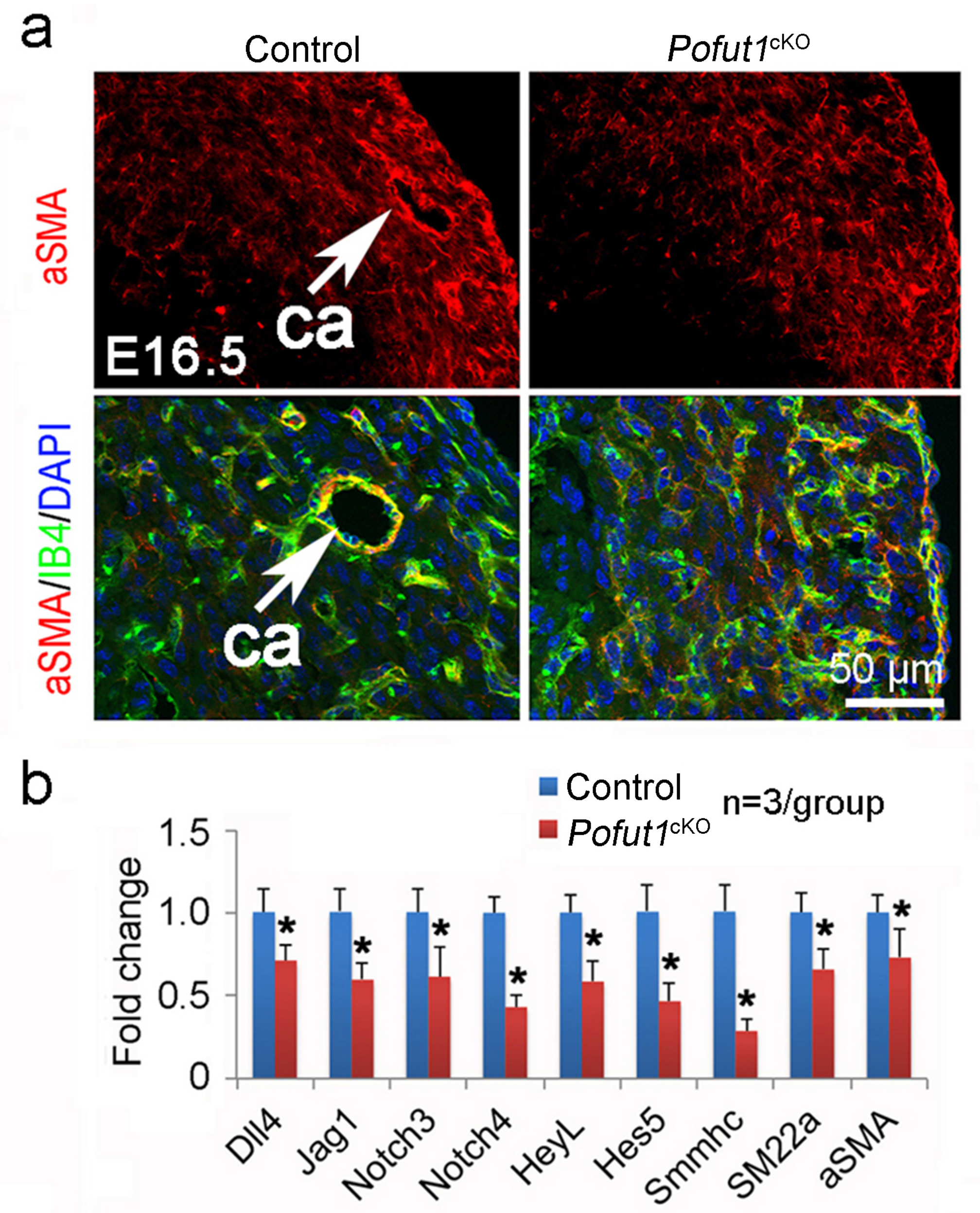

Supplementary Fig. 4. Loss of endocardial *Pofut1* causes coronary artery defects. (**a**) Co-immunostaining Isolectin-B4 (IB4) (green) with αSMA (red) showing hypoplastic major coronary arteries with less smooth muscle coverage. Arrows indicate mature coronary arteries (ca). (**b**) qPCR analysis of coronary defects in E16.5 *Pofut1*^{cKO} embryos shows decreased expression of NOTCH pathway and vascular smooth muscle genes. n = 3/group, mean ± SD. *$p < 0.05$.

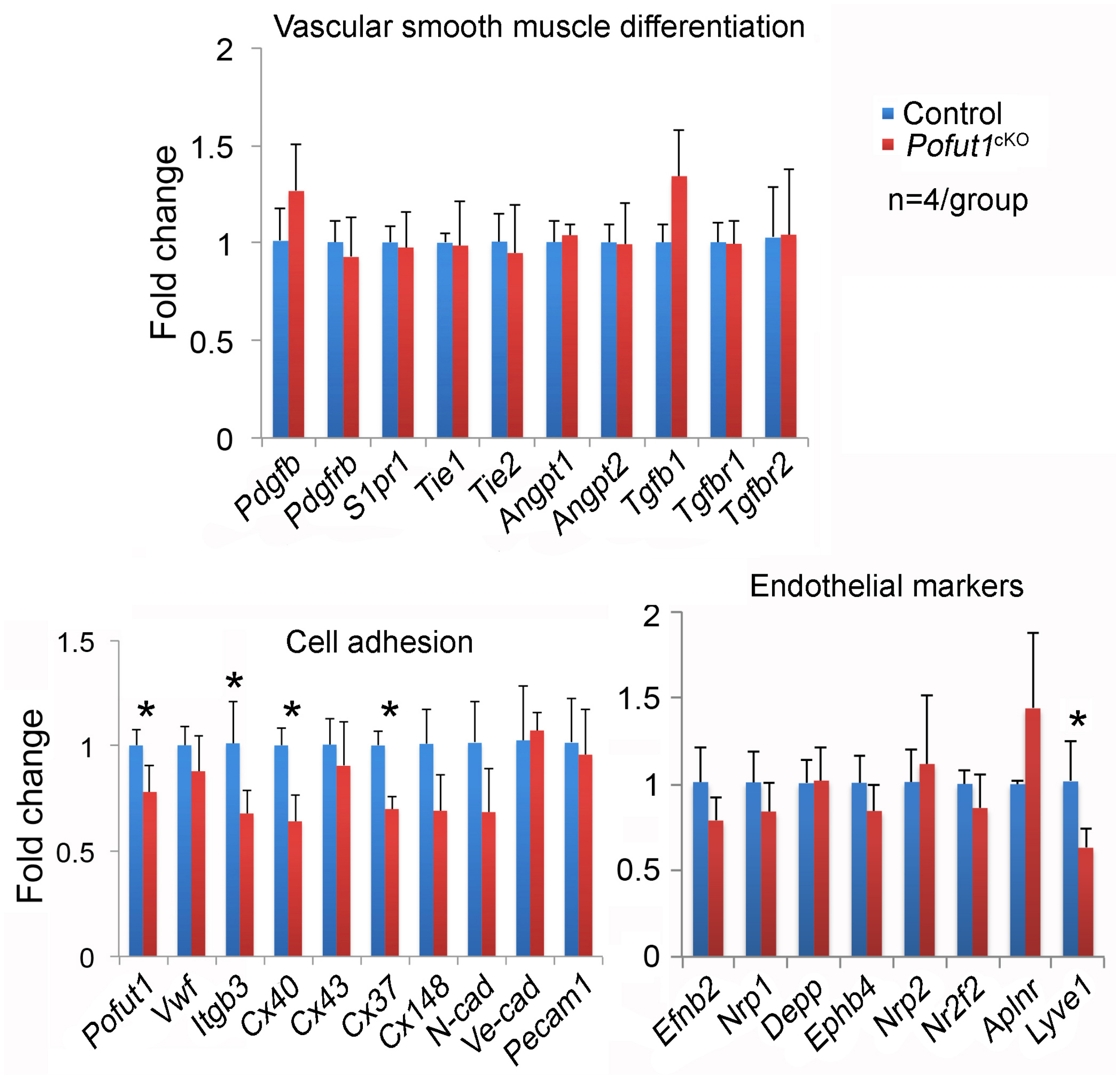

Supplementary Fig. 5. qPCR analysis of coronary defects in E16.5 *Pofut1*^{cKO} embryos. qPCR analysis showing downregulation of genes involving cell-cell adhesion in *Pofut1*^{cKO} hearts. No significant changes were found in the expression of genes related to differentiation of vascular smooth muscle cells. The arterial and venous markers were not affected by *Pofut1* deletion. n = 4/group, mean ± SD. **p*<0.05.

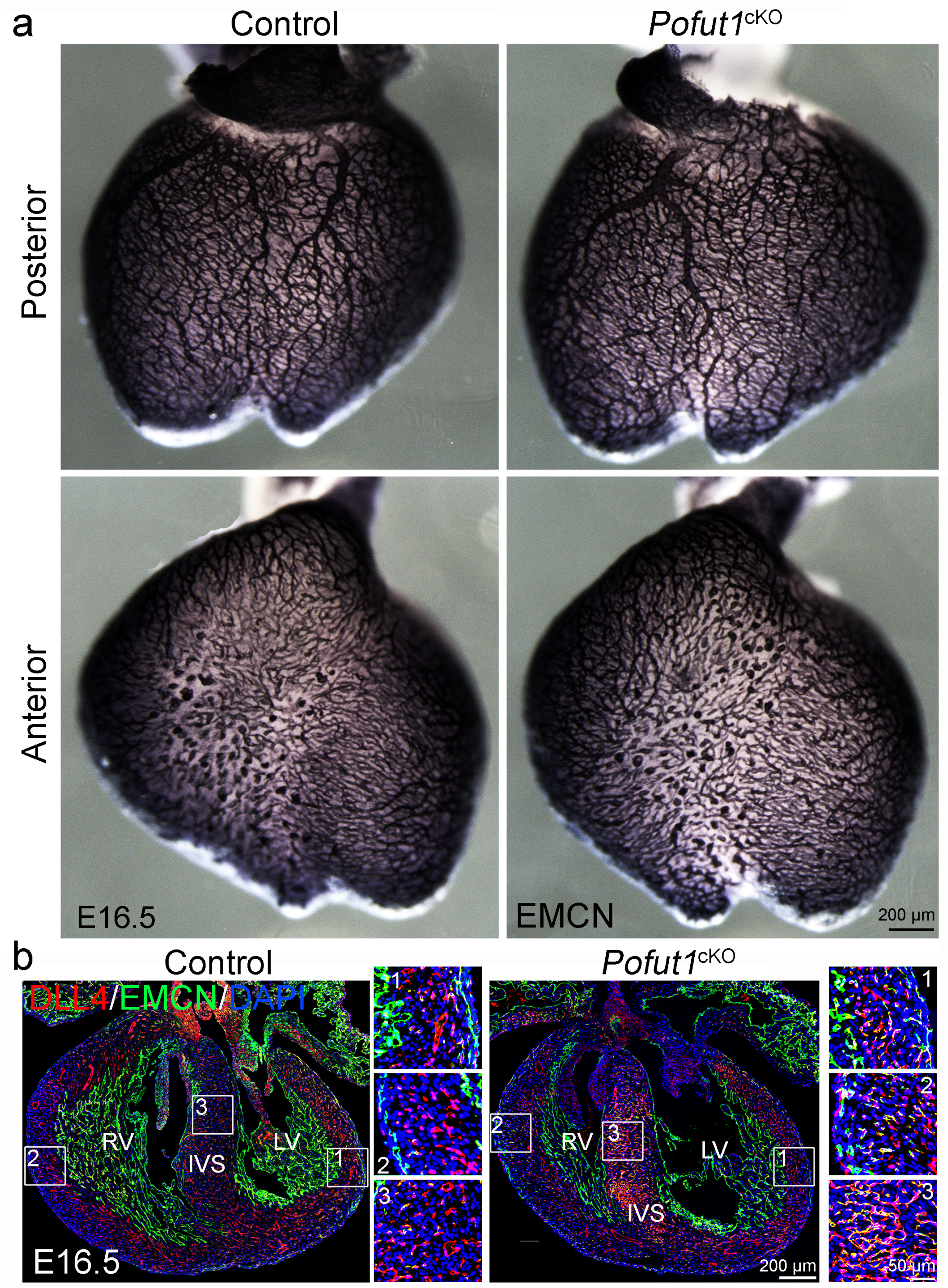

Supplementary Fig. 6. Loss of endocardial *Pofut1* results in venous marker expression in coronary arteries. (**a**) whole mount staining of EMCN shows that coronary veins on heart surface are comparable between control and *Pofut1*^{cKO} embryos. (**b**) Co-immunostaining of DLL4 and EMCN on heart sections indicates that some intramyocardial arteries in *Pofut1*^{cKO} embryos express venous maker EMCN. LV/RV, left/right ventricle; IVS, interventricular septum.

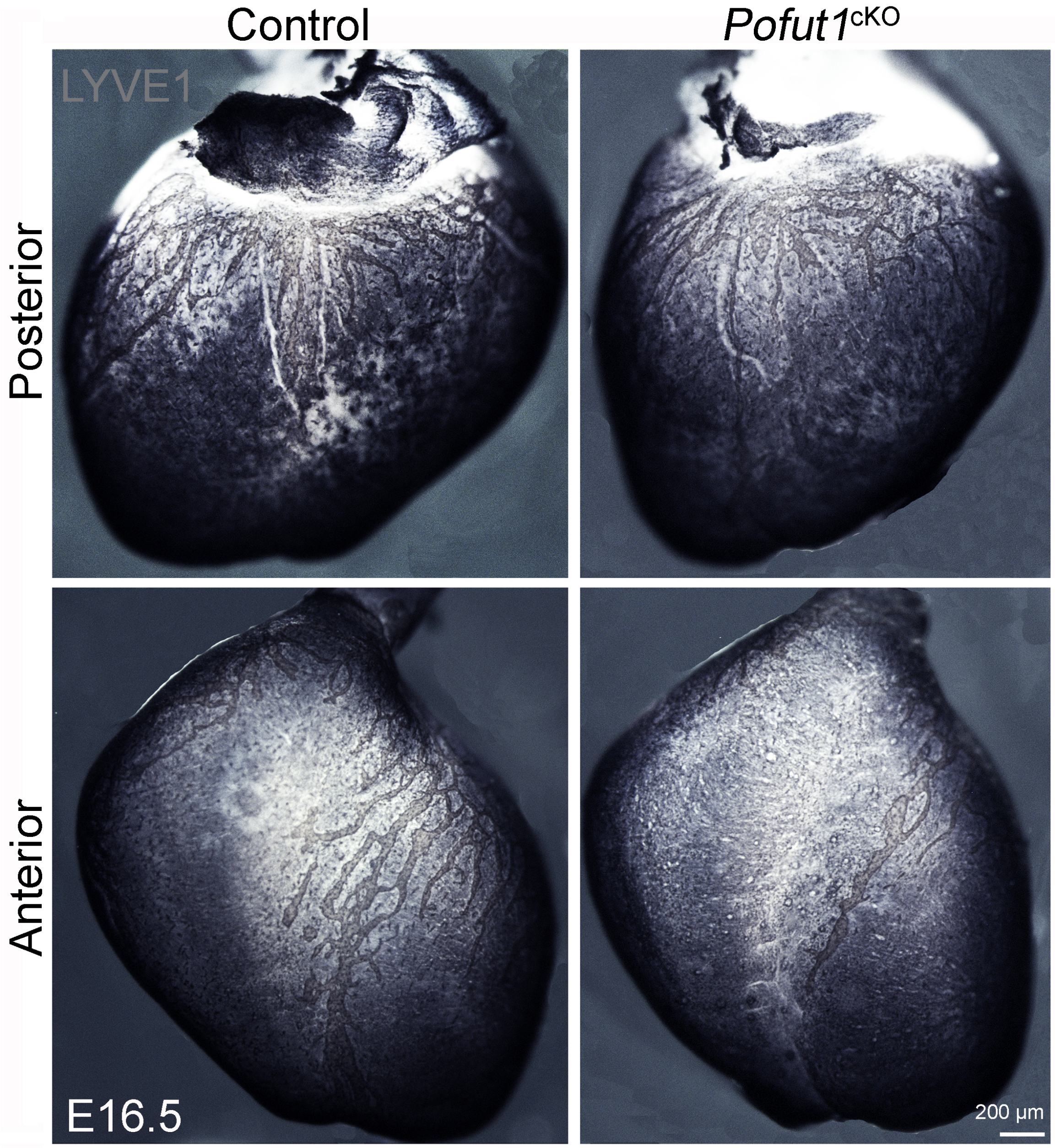

Supplementary Fig. 7. Loss of endocardial *Pofut1* causes delayed formation of cardiac lymphatic vessels. Whole mount staining of LYVE1 showing that the anterior lymphatic vessels of E16.5 *Pofut1*^{cKO} hearts are reduced as compared to controls, while the posterior lymphatic vessels are comparable between control and *Pofut1*^{cKO} hearts.

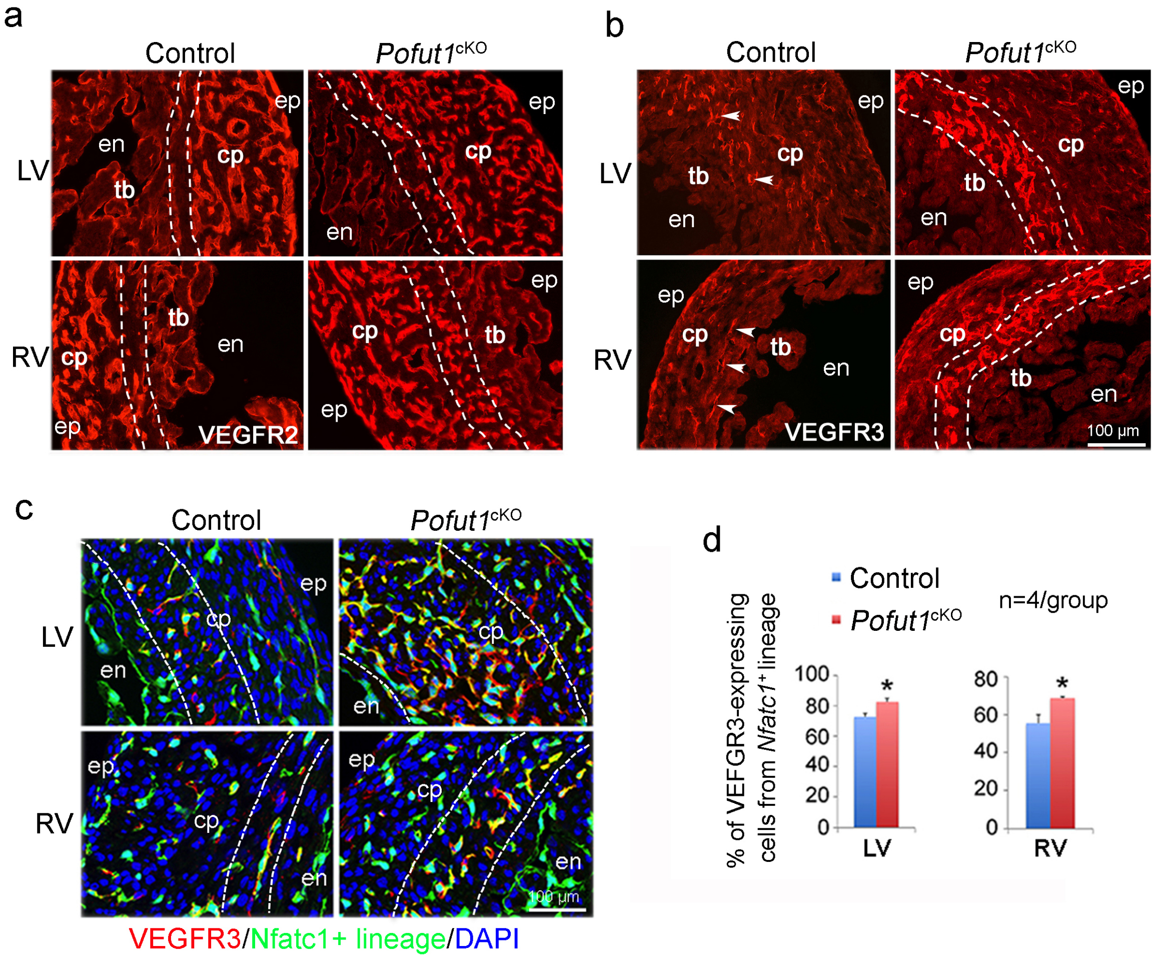

Supplementary Fig. 8. Characterization of coronary arteries of *Pofut1*^{cKO} embryos reveals a distinct coronary angiogenic cell population. (**a**) VEGFR2 staining of heart sections from E16.5 control and *Pofut1*^{cKO} embryos indicates matured coronary arteries of control heart expressing a high level of VEGFR2 in the compact myocardium (cp), whereas the endocardium of trabecular myocardium (tb) expressing a low level. Note an 'avascular' zone between cp and tb myocardium (marked by dotted lines). In *Pofut1*^{cKO} hearts this avascular zone is occupied by VEGFR2+ small vessels, and disappears. Increased density of small coronaries is seen throughout the cp myocardium. (**b**) VEGFR3 staining recognizes a population of angiogenic precursors located within the avascular zone (arrowheads) in control heart, which is greatly increased in *Pofut1*^{cKO} hearts (indicated by dotted lines). This finding suggests the 'avascular zone' as a functional 'growth zone' of coronary arteries. (**c**) GFP and VEGFR3 co-staining of heart sections from E16.5 control and *Pofut1*^{cKO} embryos shows the majority of VEGFR3-expressing coronary angiogenic cells (red staining) within the inner myocardium near ventricular endocardium (en) also expresses GFP (green staining), an lineage expressing marker for the *Nfatc1*^{Cre}-tagged endocardial cell progenies. (**d**) Quantitative analysis indicates an increased number of endocardially-derived VEGFR3+ angiogenic cells in *Pofut1*^{cKO} hearts. n = 4/group; mean ± SD; $*p < 0.05$.

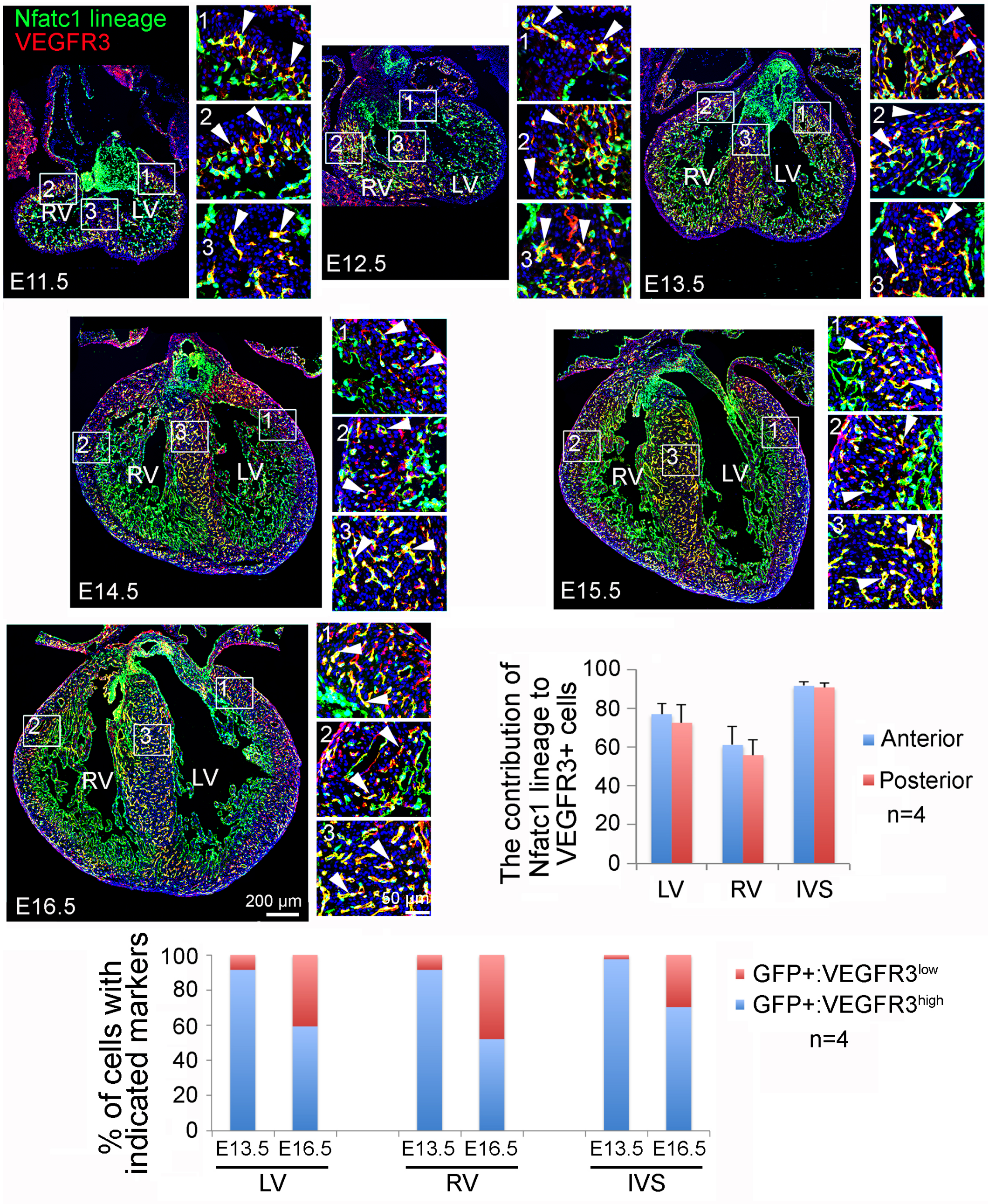

Supplementary Fig. 9. VEGFR3^{high} cells are derived from endocardium. The origin of VEGFR3^{high} cells is shown by co-immunostaining of VEGFR3 with *Nfatc1*$^{+}$ lineage marker GFP on heart sections from E11.5 to E16.5. VEGFR3 is highly expressed in endocardial cells at base of trabeculae at E11.5 when coronary plexus starts to form. The sprouting by endocardial cells (arrowhead) is present within coronary sulcus and interventricular septum. VEGFR3^{high} cell population expands in the ventricular wall and form coronary plexus after E12.5. By E15.5, VEGFR^{high} cells are mainly located in the inner myocardium close to endocardium. Quantification of VEGFR3^{high} cells at E16.5 showed that majority of VEGFR3^{high} cells (>70%) is derived from *Nfatc1*$^{+}$ lineage. At E13.5, >90% of *Nfatc1*$^{+}$-GFP cells express high levels of VEGFR3, while the percentage reduces to 60% at E16.5.

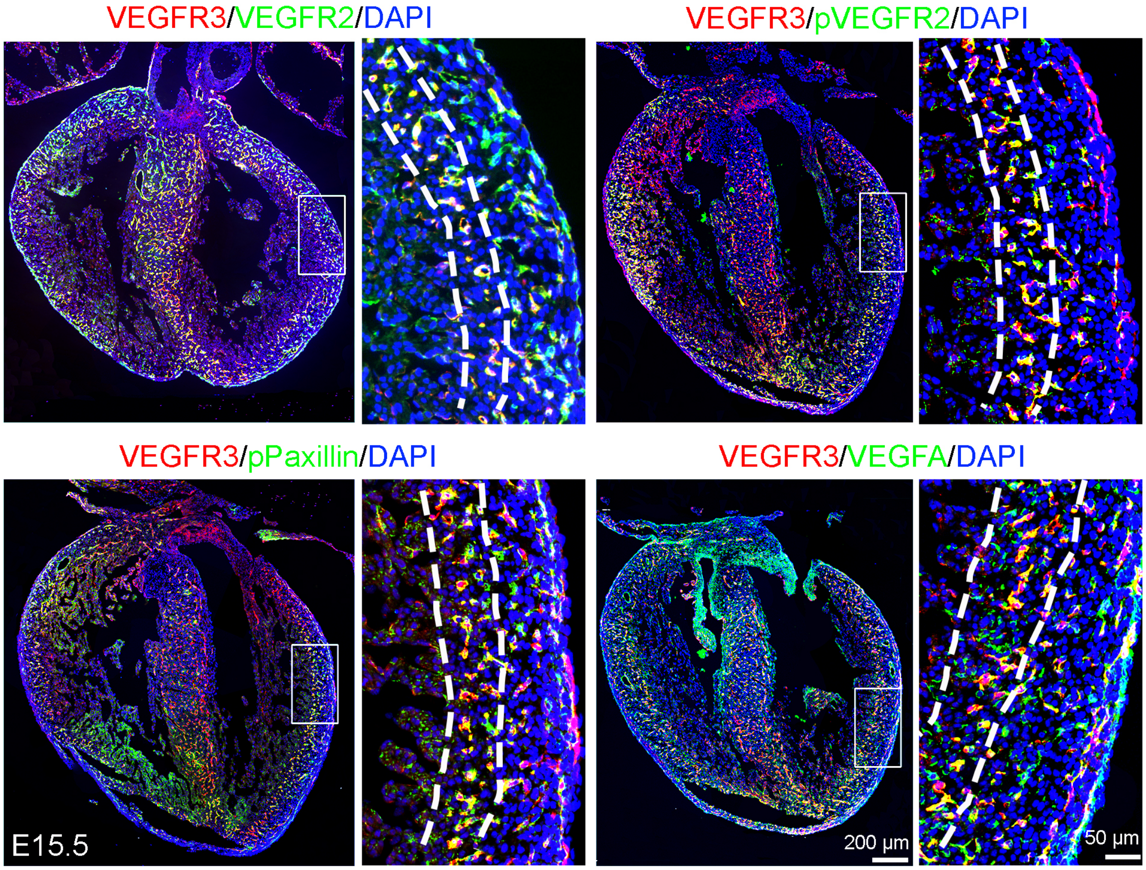

Supplementary Fig. 10. VEGFR3^{high} cells are angiogenic and migratory. VEGFR3^{high} cells are characterized by co-immunostaining of VEGFR3 with angiogenic and migratory markers on E15.5 heart sections. The results show that VEGFR3^{high} cells are a subpopulation of VEGFR2-positive cells and express high levels of pVEGFR2 and pPAXILLIN. Noted that VEGFA is enriched in the inner myocardial region (space between dotted lines) where VEGFR3^{high} cells are located.

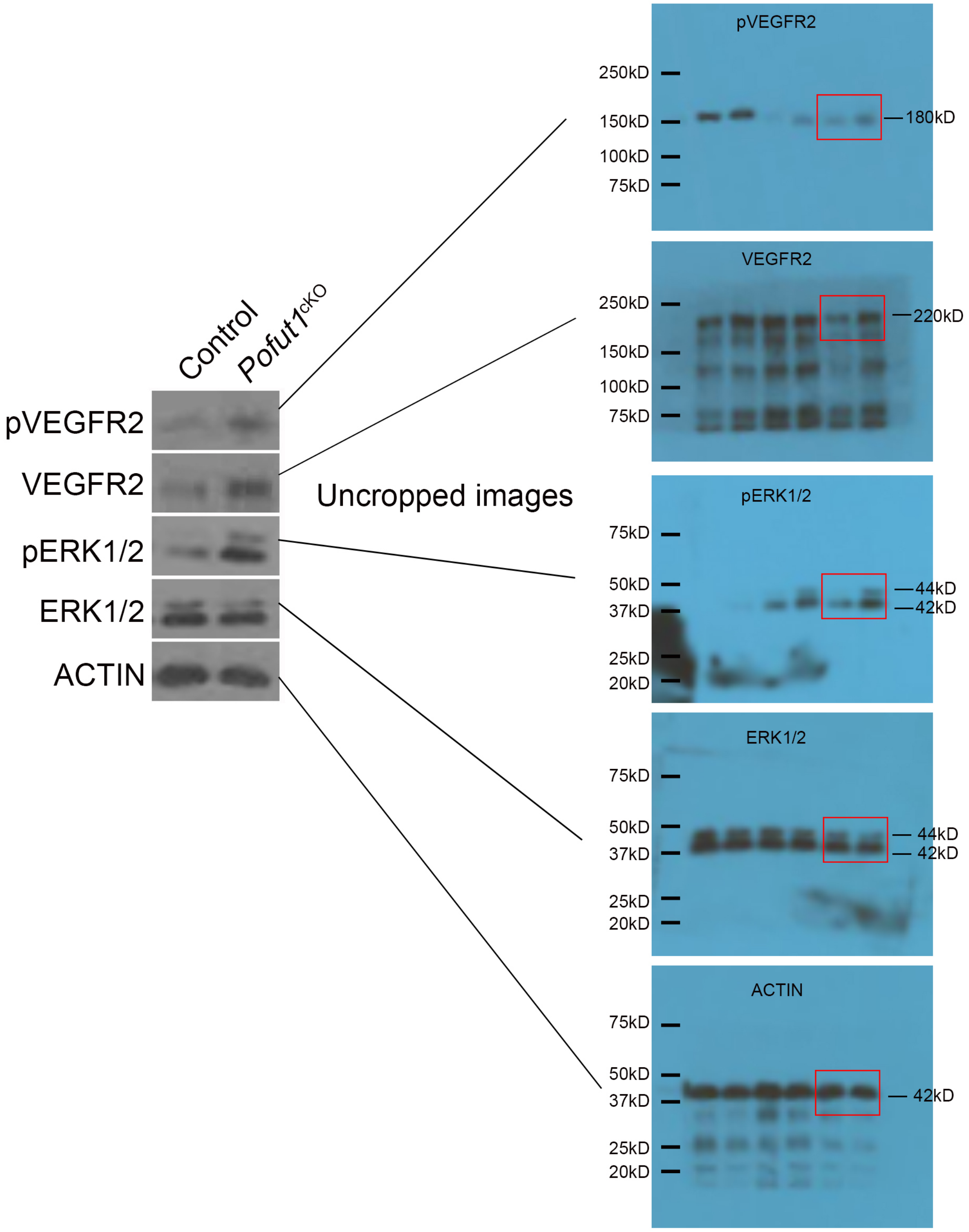

Supplementary Fig. 11. Original images of Fig. 3l. The blots in Fig. 3l showing on left panel were cropped from the original film scans showing on right panel. The red boxes indicate the cropped regions.

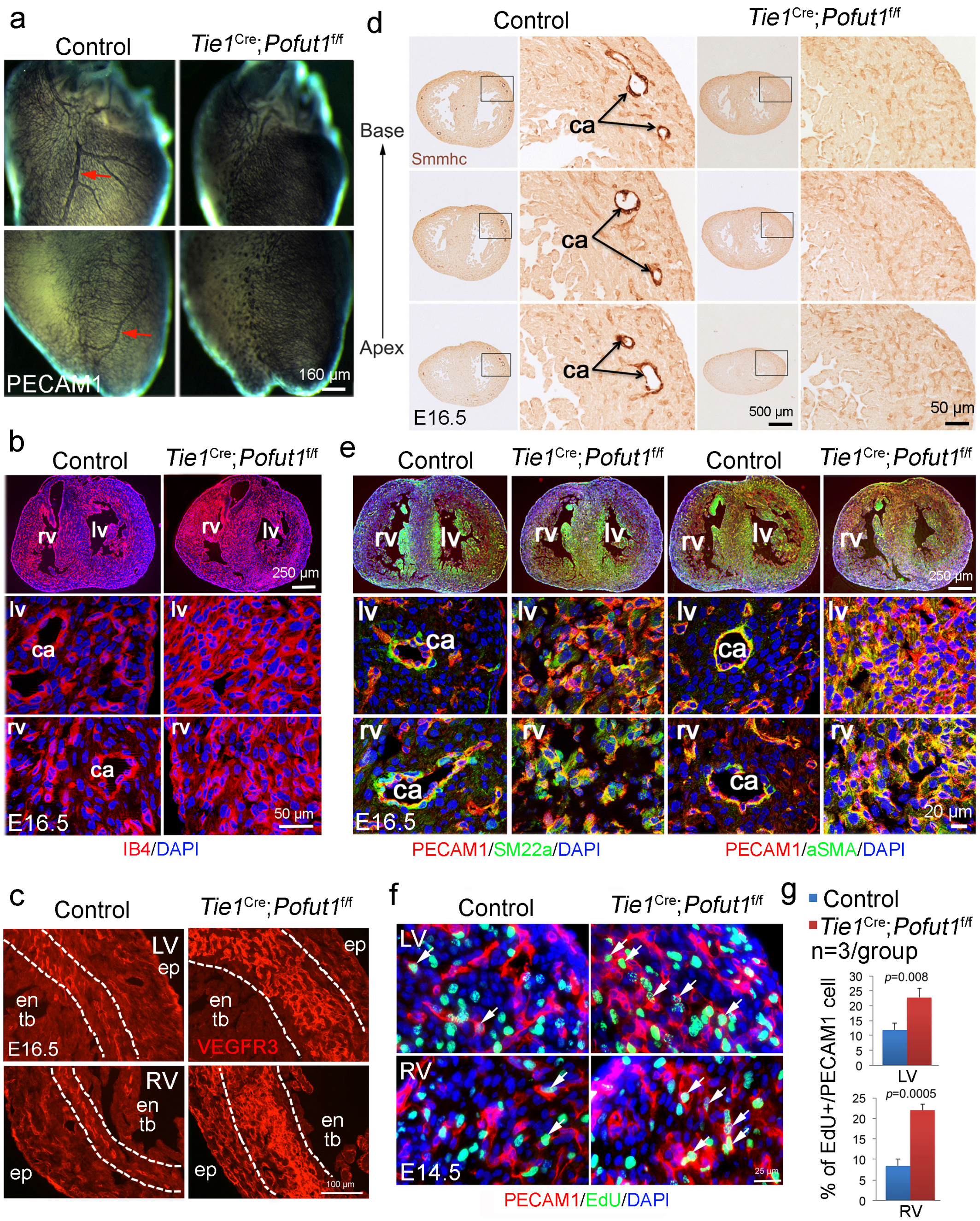

Supplementary Fig. 12. Pan-endothelial deletion of *Pofut1* results in a similar but more severe coronary artery phenotype. (**a**,**b**) Whole mount and sectional PECAM1 staining of E16.5 hearts show no formation of main coronary arteries (ca, arrow) and increased coronary networks in the heart with *Pofut1* deletion in pan-endothelium using *Tie1^{Cre}*. (**c**) VEGFR3 staining shows expansion of area of VEGFR3^{high} coronary angiogenic cells in the inner myocardium (between dotted lines) of E16.5 endothelial *Pofut1* deleted heart. (**d**) SMMHC staining shows major coronary arteries are absent in the *Pofut1* deleted heart. (**e**) PECAM1 and smooth muscle cell marker (SM22α or αSMA) staining. (**f**,**g**) EdU assay shows increased proliferation of coronary endothelial cells (arrows) in E14.5 *Pofut1* deleted heart. n = 3/group, mean ± SD.

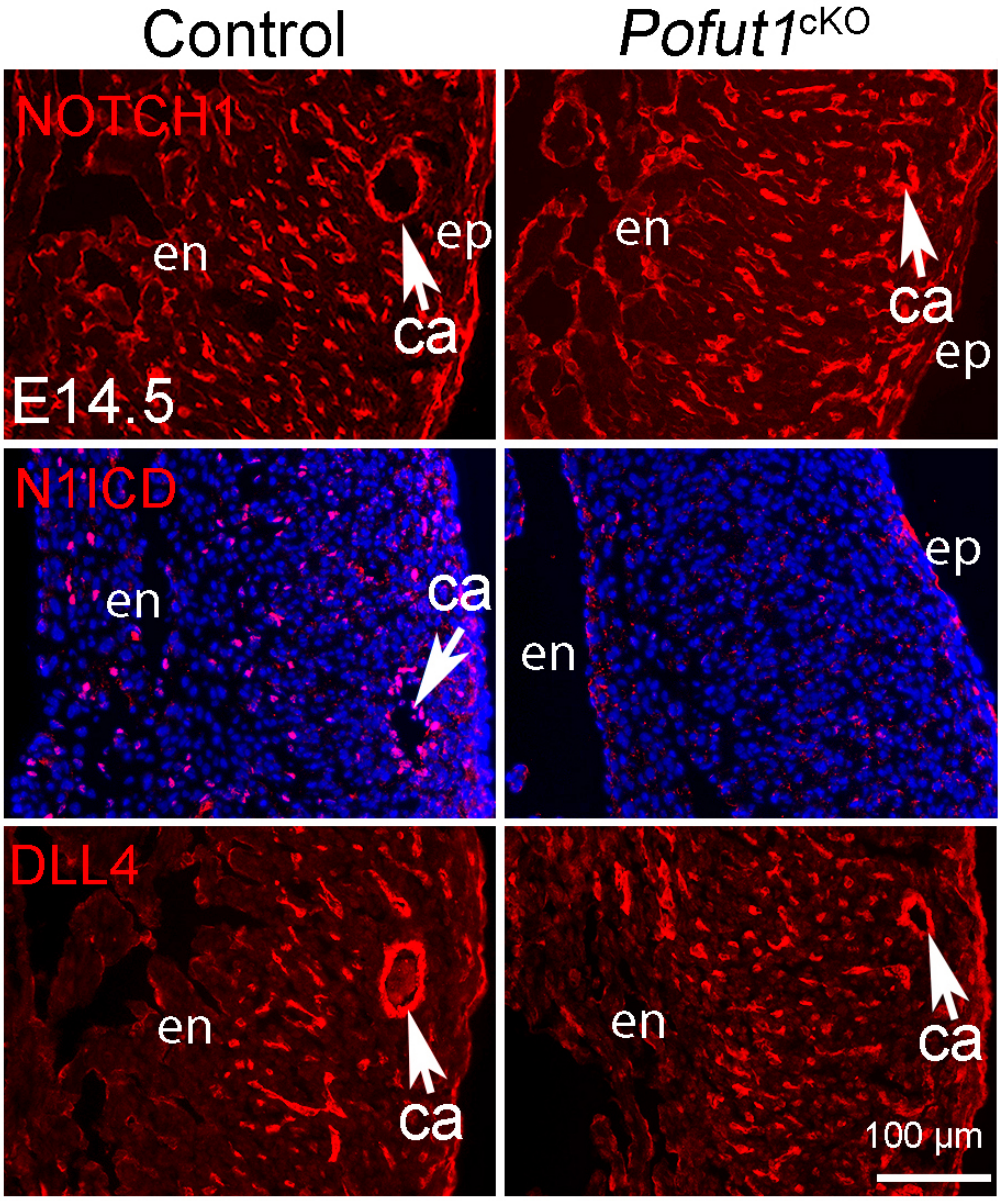

Supplementary Fig. 13. Loss of endocardial *Pofut1* causes reduced N1ICD.
(**a**) Immunostaining for membrane NOTCH1, N1ICD and DLL4 showing reduced expression of N1ICD in the coronary plexuses and coronary arteries (ca) in E14.5 *Pofut1*^{cKO} heart, while expression of membrane NOTCH1 and DLL4 is not affected. en/ep, endocardium/epicardium.

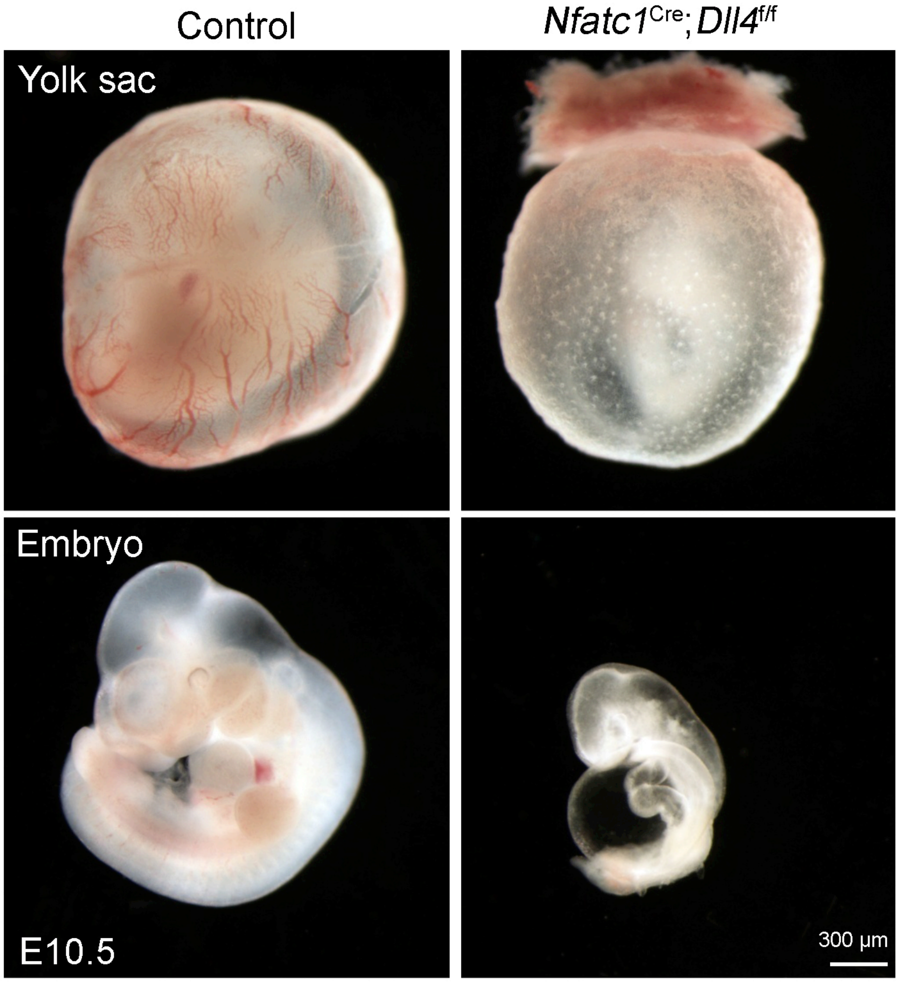

Supplementary Fig. 14. Loss of endocardial *Dll4* causes severe delay development. Whole mount views show that E10.5 *Nfatc1*^{Cre};*Dll4*$^{f/f}$ embryos have defective yolk sac vessels and are developmentally arrested at E10.5.

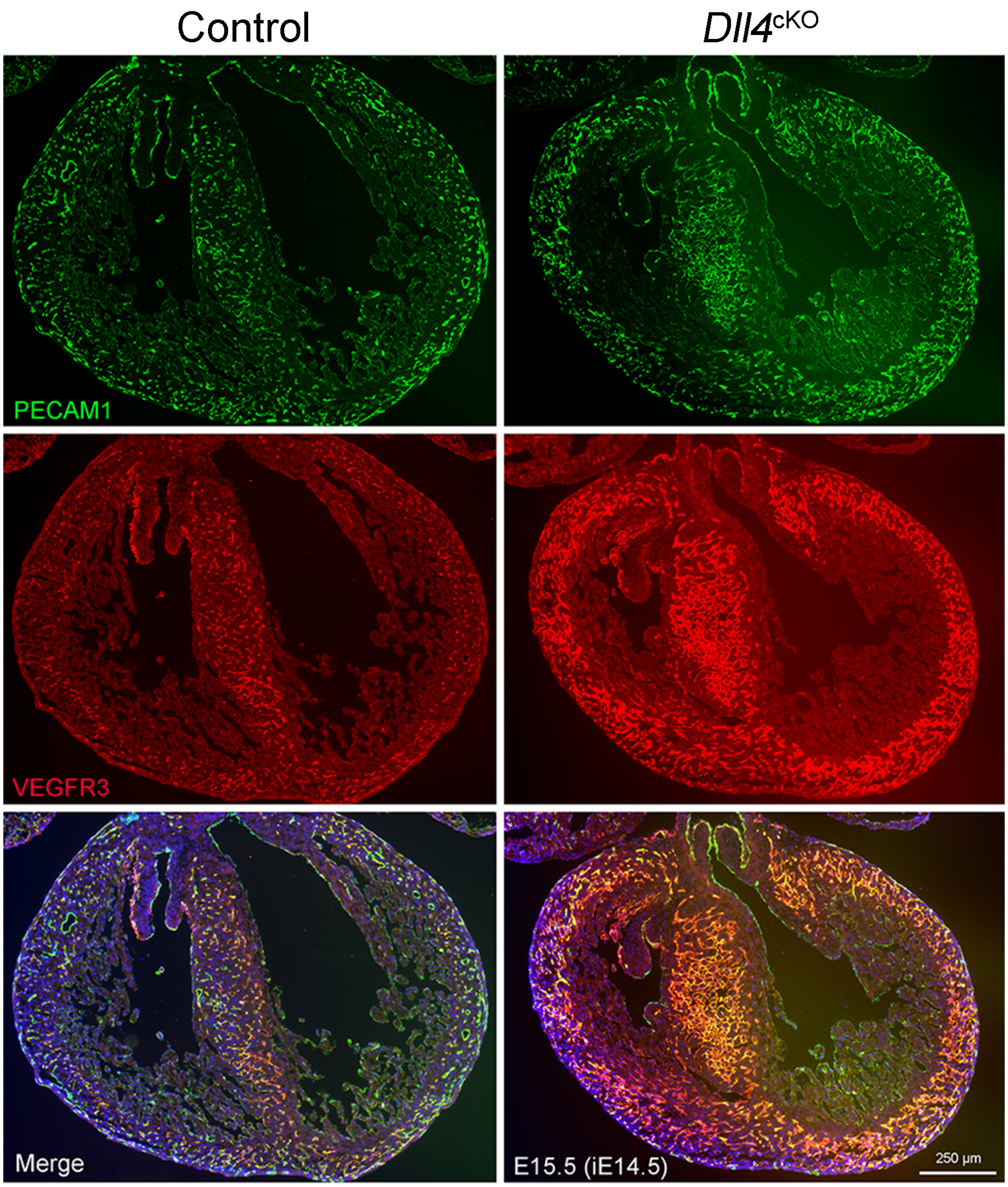

Supplementary Fig. 15. Deletion of *Dll4* results in a similar coronary phenotype seen in *Pofut1*^{cKO} embryos. PECAM1 and VEGFR3 co-staining E15.5 control and *Dll4*$^{f/f}$;*Cdh5*^{CreERT2} (*Dll4*^{cKO}) embryos shows that induced *Dll4* deletion at E14.5 results in increased coronaries at E15.5 which express VEGFR3.

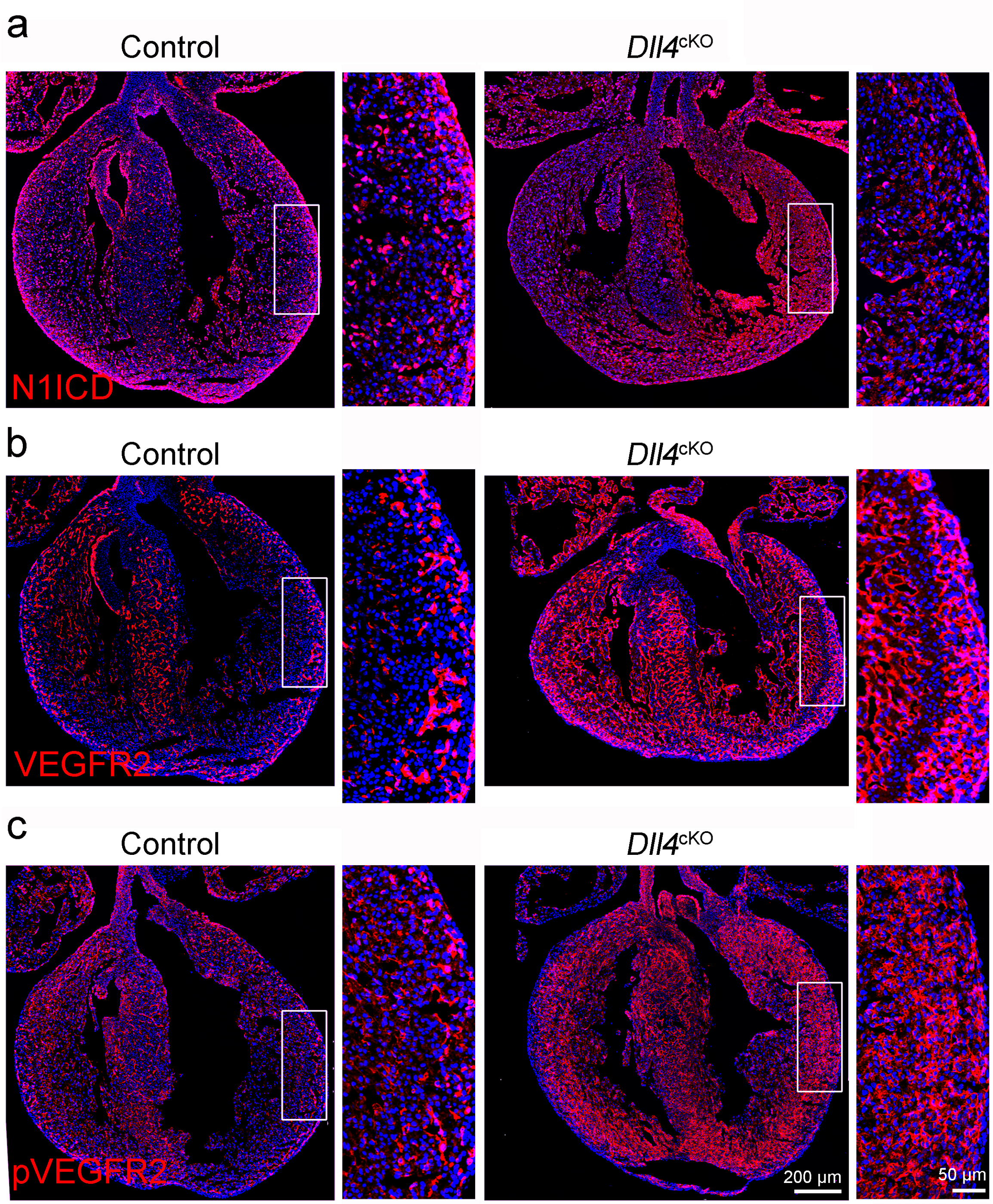

Supplementary Fig. 16. Loss of *Dll4* decreases expression of N1ICD and increases expression of VEGFR2 and pVEGFR2. (a-c) Immunostaining with indicated antibodies on heart sections from E15.5 control and $Dll4^{f/f};Cdh5^{CreERT2}$ ($Dll4^{cKO}$) embryos shows that deletion of *Dll4* at E14.5 decreases levels of N1ICD and increases levels of VEGFR2 and pVEGFR2.

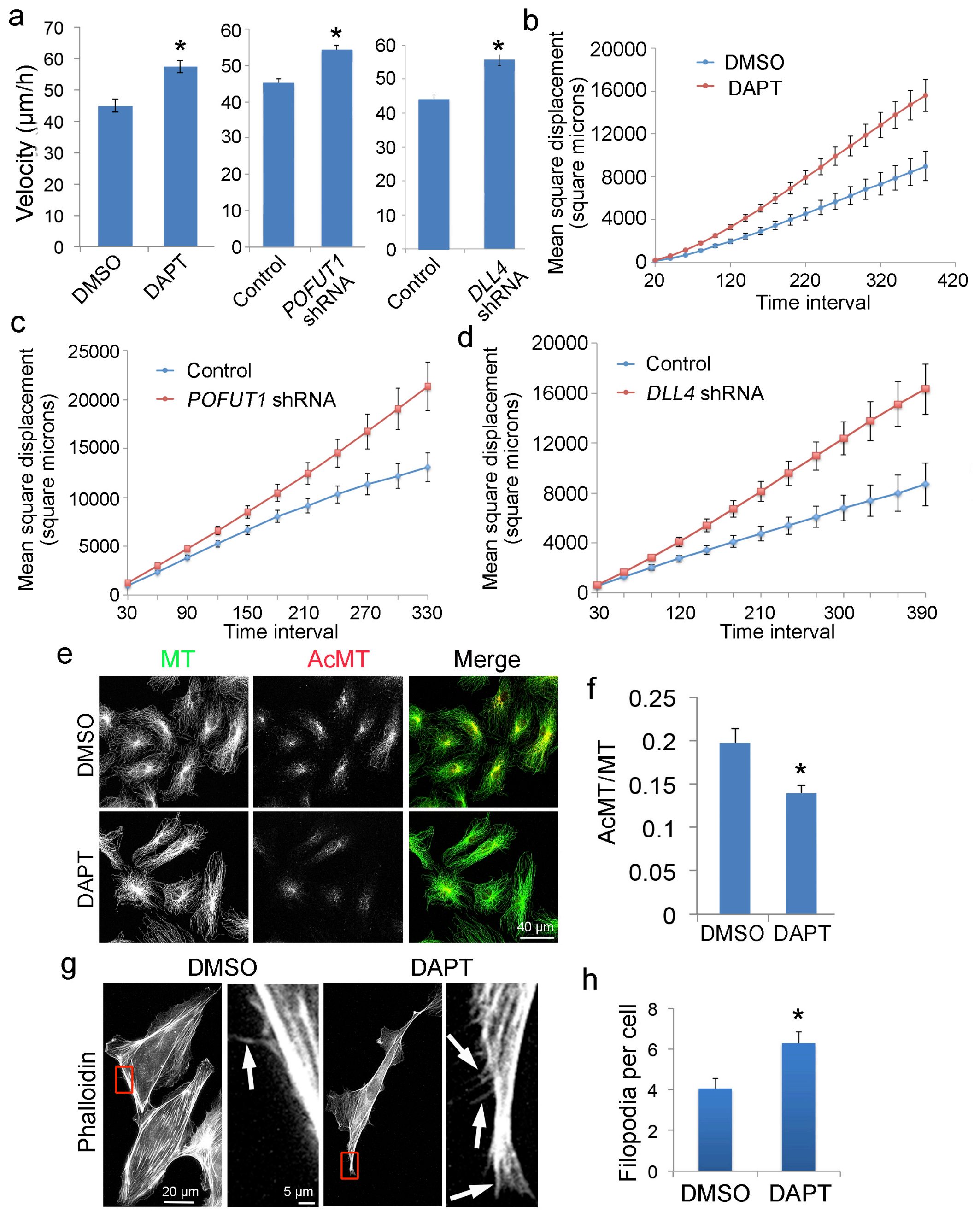

Supplementary Fig. 17. Inhibition of NOTCH promotes endothelial cell migration.
(**a-d**) Quantification of time-lapse images shows that inhibition of NOTCH by DAPT significantly promotes cell mobility indicating by increased velocity and mean square displacement. Knockdown of *POFUT1* or *DLL4* with shRNA promotes cell migration indicating by increased velocity and mean square displacement. (**e**,**f**) Co-staining for microtubules (MT) and acetylated microtubules (AcMT) shows that inhibition of NOTCH reduces the ratio of AcMT to total MT, an indicative of increased cell mobility. (**g**,**h**) Phalloidin staining shows an increased number of filopodia by inhibition of NOTCH. n = 100 cells/group, mean ± SD; * $p < 0.05$.

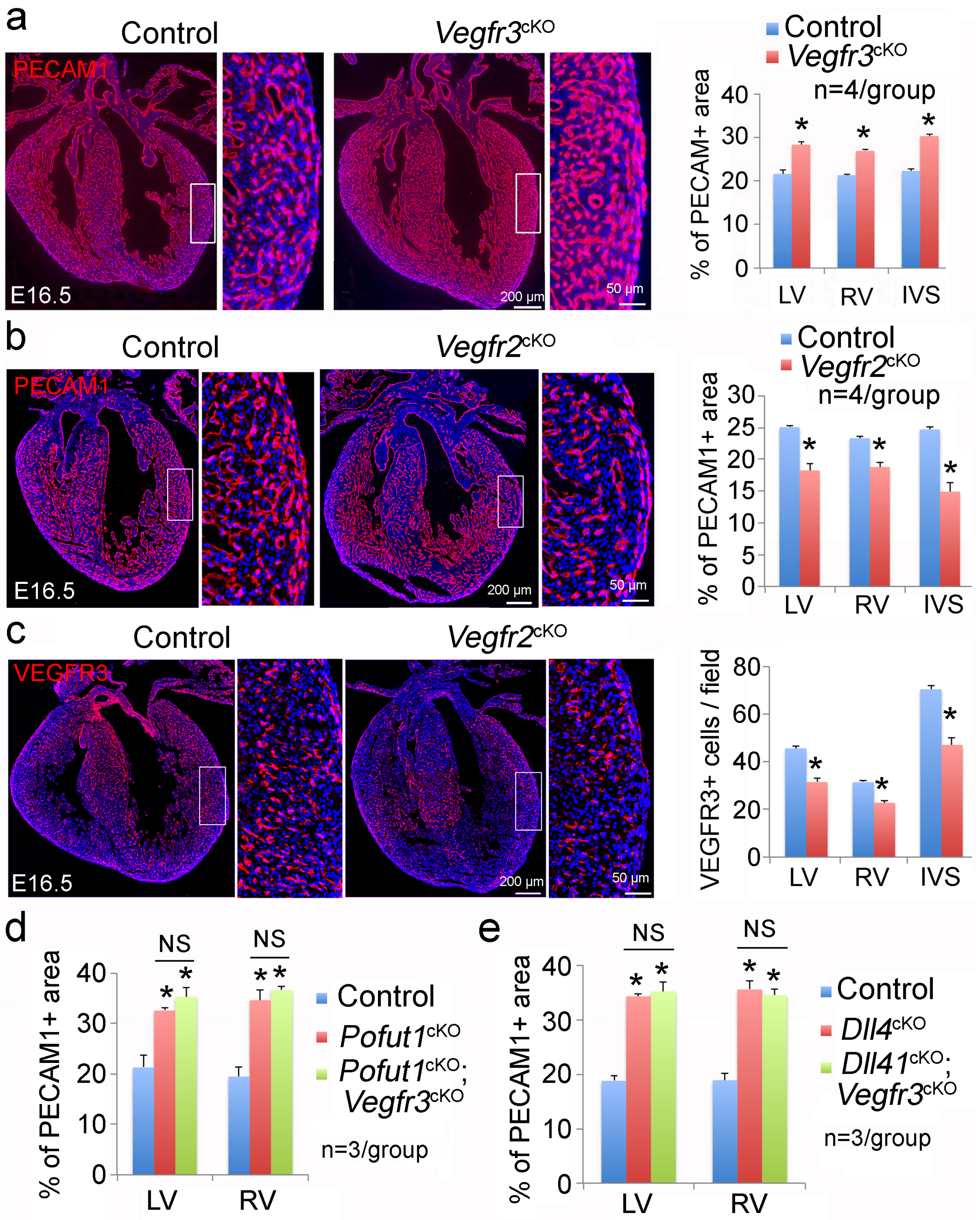

Supplementary Fig. 18. VEGFR3 and VEGFR2 have opposite roles in coronary vasculogenesis. (**a**,**b**) PECAM1 immunostaining shows that *Vegfr3*$^{f/f}$;*Nfatc1*^{Cre} (*Vegfr3*^{cKO}) embryos have increased coronary vessels, whereas *Vegfr2*$^{f/f}$;*Nfatc1*^{Cre} (*Vegfr2*^{cKO}) embryos have decreased coronary vessels. (**c**) VEGFR3 immunostaining shows that deletion of *Vegfr2* results in reduced number of VEGFR3-positive cells. (**d**,**e**) Quantification of PECAM1-stained cells in Fig. 5 suggests that deletion of *Vegfr3* is not able to rescue the coronary defects resulting from deletion of *Pofut1* or *Dll4*.

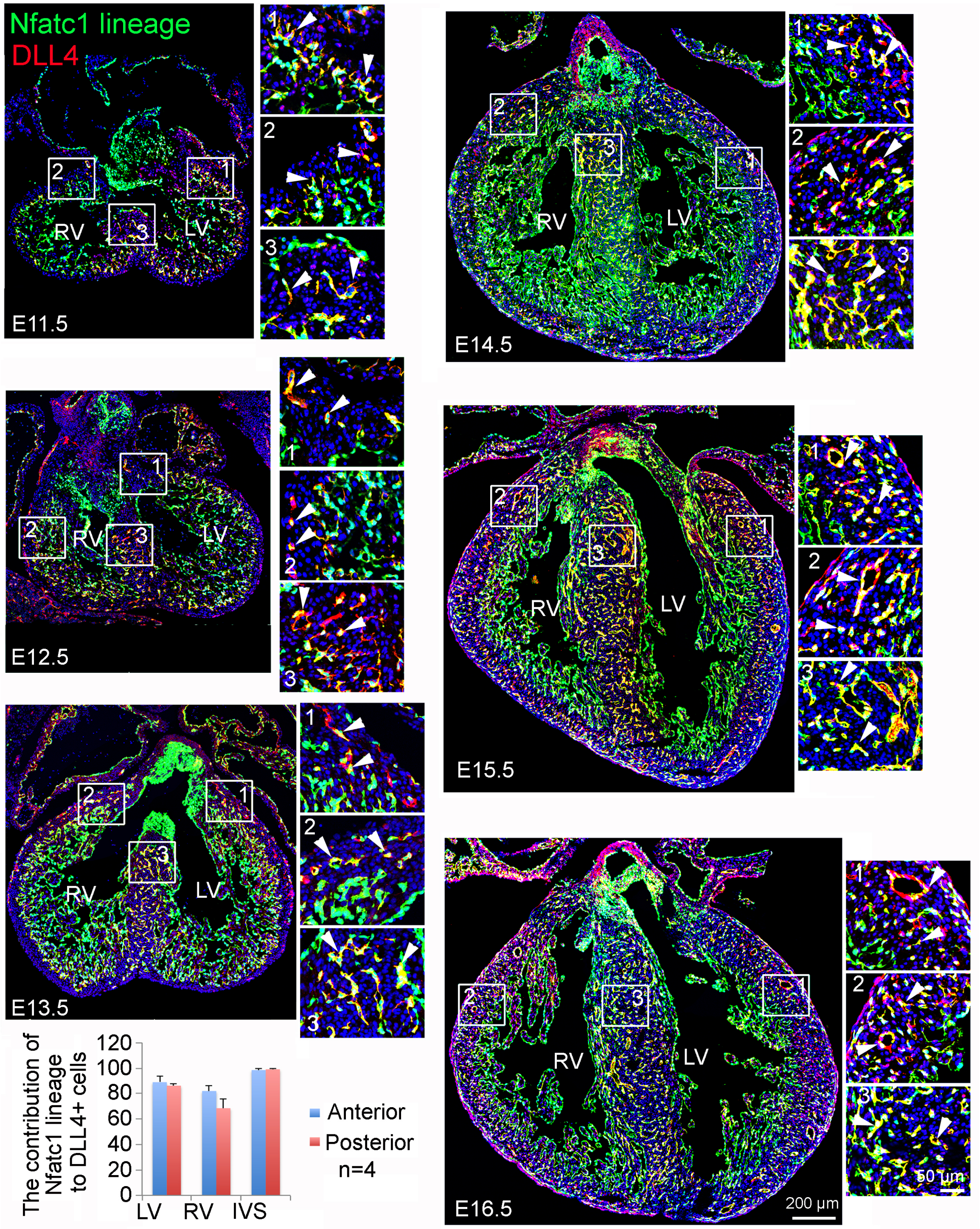

Supplementary Fig. 19. *Nfatc1*⁺ lineage has a major contribution to coronary arterial endothelium. Contribution of *Nfatc1*⁺ lineage to coronary arterial endothelium is revealed by co-immunostaining of DLL4 (arrows) with *Nfatc1*⁺ lineage marker GFP on heart sections, LV/RV, left/right ventricle. Quantitative results indicate that *Nfatc1*⁺ lineage has a major contribution to coronary arterial endothelium.

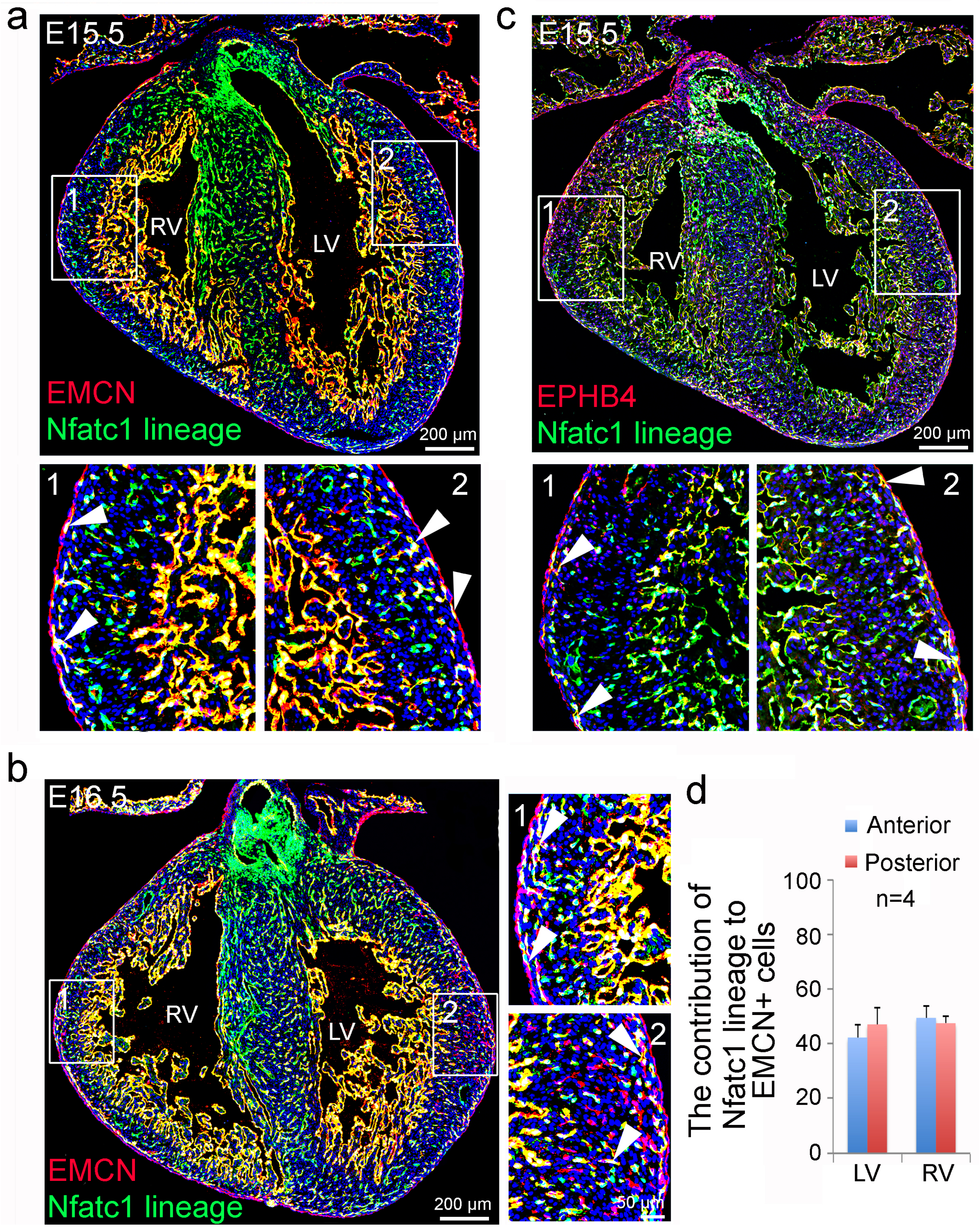

Supplementary Fig. 20. *Nfatc1*$^{+}$ lineage has a minor contribution to coronary venous endothelium. (**a-d**) contribution of *Nfatc1*$^{+}$ lineage to venous endothelium is viewed by co-immunostaining of EMCN or EPHB4 (arrows) with *Nfatc1*$^{+}$ lineage maker GFP on heart sections from embryos at indicated stages. LV/RV, left/right ventricle. Quantitative results show that *Nfatc1*$^{+}$ lineage has a minor contribution to coronary venous endothelium.

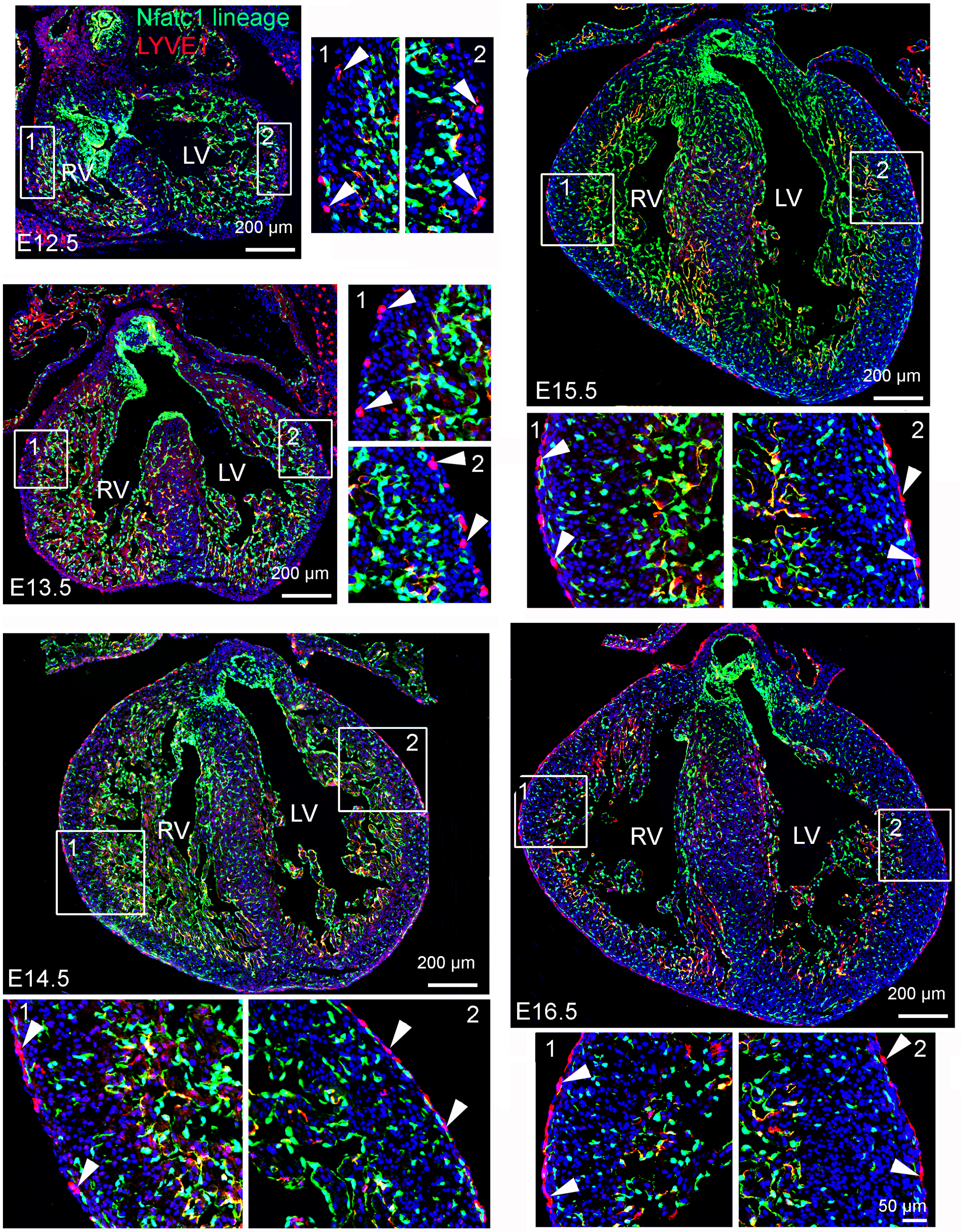

Supplementary Fig. 21. ***Nfatc1***$^{+}$ **lineage has no or very little contribution to cardiac lymphatic vessels.** Contribution of *Nfatc1*$^{+}$ lineage to lymphatic vessels is viewed by co-immunostaining of LYVE1 (arrows) with *Nfatc1*$^{+}$ lineage maker GFP on heart sections from E12.5 to E16.5 embryos. LV/RV, left/right ventricle.

Cardiac lineages	Cre drivers	Gross phenotype	# of mice analyzed
Pan-endothelial cells	*Tie1*^{Cre}	Embryonic lethal before birth	45
Endocardial lineage	*Nfatc1*^{Cre}	80% of mice died by 4-month	50
Cardiomyocytes	*Tnt*^{Cre}	Normal gross morphology and behavior by 4-month	18
Secondary heart field	*Mef2c*^{Cre}	Normal gross morphology and behavior by 4-month	16
Epicardial lineage	*Tbx18*^{Cre}	Normal gross morphology and behavior by 4-month	13
Smooth muscle cells	$Sm22\alpha^{Cre}$	Normal gross morphology and behavior by 4-month	15

Supplementary Table 1. Summary of the survival of mutant mice with deletion of *Pofut1* in individual cardiac tissues. PCR genotyping results show the lethal phenotypes of *Pofut1* deletion in pan-endothelium or endocardium.

	Mean fluorescence intensity of DLL4 (n=3)	Mean fluorescence intensity of NOTCH1 (n=3)
Control	775 ± 19	182 ± 11
Pofut1 KO clone1	14 ± 2	126 ± 12
Pofut1 KO clone 2	16 ± 3	144 ± 12

Supplementary Table 2. Loss of *Pofut1* impairs DLL4 binding. DLL4-Fc binding assays in CHO cells show that loss of *Pofut1* dramatically blocked the binding of DLL4. Immunostaining using an antibody against extracellular NOTCH1 shows that loss of *Pofut1* has minor effects on the levels of cell membrane NOTCH1. The experiment was performed in two *Pofut1* knockout clones.

Name	Manufacturer / Cat#	Dilutions
POFUT1	Santa Cruz Biotechnology (sc271026)	(1:100)
EMCN	Santa Cruz Biotechnology (sc65495)	(1:100)
PAXILLIN (phospho)	Cell signaling (#2541)	(1:100)
PECAM1	BD Pharmingen (550274)	(1:100)
VEGFA	Abcam (ab46154)	(1:100)
VEGFR2	BD Pharmingen (550549)	(1:100)
VEGFR2 (phospho)	Abcam (ab5473)	(1:100)
VEGFR3	R&D (AF743)	(1:100)
NOTCH1	R&D (AF5267)	(1:100)
NOTCH1 (N1ICD)	Cell signaling (#4147)	(1:100)
DLL4	R&D (AF1389)	(1:100)
SMMHC	BTI (BT-562)	(1:300)
α-SMA	Abcam (ab32575)	(1:400)
SM22α	Abcam (ab14106)	(1:200)
LYVE1	Abcam (ab14917)	(1:100)
EPHB4	R&D (AF446)	(1:100)
Isolectin-B4	Sigma (L-2140)	(1:50)
Donkey anti-sheep IgG (Alexa Fluor 568)	Life techology (A21099)	(1:200)
Goat anti-Rat IgG (Alexa Fluor 568)	Life techology (A11077)	(1:200)
Donkey anti-Rat IgG (Alexa Fluor 488)	Life techology (A21208)	(1:200)
Donkey anti-Goat IgG (Alexa Fluor 568)	Life techology (A11057)	(1:200)
Goat anti-Rabbit IgG (Alexa Fluor 568)	Life techology (A11036)	(1:200)
Goat anti-Rabbit IgG (Alexa Fluor 488)	Life techology (A11034)	(1:200)
Goat anti-Mouse IgG (Alexa Fluor 488)	Life techology (A11017)	(1:200)

Supplementary Table 3. Summaries of antibodies used in the study. Antibody information and working condition in immunostaining are provided in the table.

Gene	Primer sequence (5' to 3')	Size (bp)	Gene	Primer sequence (5' to 3')	Size (bp)
Angpt1	GCGCTGGCAGTACAATGACAGTTT<br>CACATTGCCCATGTTGAATCCGGT	138	*Nr2f2*	TTCCTGGTTTGGGAAGAGCTCACT<br>AAGTATTGTCAGGGAGGGCAGCTT	85
Angpt2	AGAAAGTTCTGGACATGGAGGGCA<br>TCTCCATTAGGTCATGCTGCTGCT	183	*Notch3*	TGCAGTCAGCTGAGAATGACCACT<br>ACATCCCGAAGTGGGTATGGGAAA	112
Aplnr	TTTGGGAAAGAGTGACTGGAGCCT<br>TGTCCAGAGCCCTCCAAGAACAAA	120	*Notch4*	AAGACATGGACGAGTGTAGCAGCA<br>TCTTTCTCACAGTGTGGCCCTTCA	118
aSMA	ATTGTGCTGGACTCTGGAGATGGT<br>TGATGTCACGGACAATCTCACGCT	187	*Pofut1*	CCCAGGAAAGTGCTAACTGAA<br>TCAGCACCTAGAAGTTGGAAAG	121
Cx37	TGCTCTTCATCTTCCGCATCCTCA<br>AAGAGGAACTGCAGCACCCAGTAT	169	*Pecam1*	TTGAGCCTCACCAAGAGAACGGAA<br>AATCCAGGAATCGGCTGCTCTTCT	114
Cx40	ATCTCTGTGTGCCTCTGTGTGTGT<br>TTAAGAAGCAAGCTCAGCCCTCCT	155	*Pdgfb*	TGTGTCTTCTTCCTCATGTGCCCT<br>TCCCATTACAACCTTGCTCACCCT	155
Cx43	TCATCTTCATGCTGGTGGTGTCCT<br>TGGTGAGGAGCAGCCATTGAAGTA	194	*Pdgfrb*	ACTACATCTCCAAAGGCAGCACCT<br>TGTAGAACTGGTCGTTCATGGGCA	177
CD148	GTGACAAATGTCAGCACAAGGGCA<br>TCACCTCCGTCCTGCTTGATGAAA	92	*S1pr1*	TGACCTTCCGCAAGAACATCTCCA<br>AGCAGGCAATGAAGACACTCAGGA	102
Depp	AAACCACAGCACATCGTCCTGACT<br>TTTCCCGAATCGTTGGCAAATGGG	199	*Smmhc*	TGAGCTCAGTGACAAGGTCCACAA<br>GGAAGCCACATCTTTGGCCAGTTT	109
Dll4	TCGAAATGGTGGCAGCTGTAAGGA<br>ATGCTCACAGTGCTGGCCATAGTA	85	*Sm22a*	TCTAATGGCTTTGGGCAGTTTGGC<br>TTTGAAGGCCAATGACGTGCTTCC	149
EfnB2	TTCTGCTGGATCAGCCAGGAATCA<br>TCCTGATGCGATCCCTGCGAATAA	196	*Tie1*	CAGCATGAAACTTCGCAAGCCAGA<br>TTTGACATTGACCTTGAAGCGCCG	184
EphB4	TCTCAGCAAAGCTGGCTTCTACCT<br>TGATCAGCCAGGAGCACTTCTTGT	101	*Tie2*	TCTGTGGAGTCAGCTTGCTCCTTT<br>TGAGGGATGTTTCGGCATCAGACA	102
HeyL	AGCATAGTCCCAATCCCACCATGT<br>TGGTTGTGGGAAGTCAGCTCAGAA	195	*Tgfb1*	GTGCGGCAGCTGTACATTGACTTT<br>TGTACTGTGTGTCCAGGCTCCAAA	127
Hes5	TCCTTTGTATGGGTGGGTGCATGT<br>TTCAGAACAGCCTGTGTTCTCCCA	88	*Tgfbr1*	TGTCAAAGTCAGTCCGTTGGGTCT<br>ACTTTGAAAGCCACACAAGCCCTG	111
Jag1	TAGTGAATGTGCCCTGGTGTCCAT<br>ATGATCCTAAGGCTGCCATCACCA	128	*Tgfbr2*	AGATGGCTCGCTGAACACTACCAA<br>AGAATCCTGCTGCCTCTGGTCTTT	100
N-cad	ATGGCCTTTCAAACACAGCCACAG<br>ACAATGACGTCCACCCTGTTCTCA	121	*Vwf*	TCATCGCTCCAGCCACATTCCATA<br>AGCCACGCTCACAGTGGTTATACA	189
Nrp1	TGGGAAGATTGCACCTTCTCCTGT<br>AGAACATTCGGGCCCTCTCTTGAA	133	*Ve-cad*	TAGCAAGAGTGCGCTGGAGATTCA<br>ACACATCATAGCTGGTGGTGTCCA	89
Nrp2	TCCTTAGCTTGCTCCCTCTTTGCT<br>TCTTCCTGGTGGTGTCTTCTGCAA	156			

Supplementary Table 4. List of primers used for qPCR in the study. Sequence of primers and size of PCR products are provided in the table.
